# Percutaneous Mitral Valve Repair versus Optimal Medical Therapy in Patients with Functional Mitral Regurgitation: A Systematic Review and Meta-Analysis

**DOI:** 10.1155/2019/2753146

**Published:** 2019-04-21

**Authors:** Muhammad Uzair Lodhi, Muhammad Shariq Usman, Tariq Jamal Siddiqi, Muhammad Shahzeb Khan, Muhammad Arbaz Arshad Khan, Safi U. Khan, Intekhab Askari Syed, Mustafa Rahim, Srihari S. Naidu, Rami Doukky, Mohamad Alkhouli

**Affiliations:** ^1^Department of Internal Medicine, Raleigh General Hospital, West Virginia University, WV, USA; ^2^Department of Internal Medicine, Civil Hospital, Dow University, Karachi, Pakistan; ^3^Department of Internal Medicine, John H Stroger Jr. Hospital of Cook County, Chicago, IL, USA; ^4^Department of Internal Medicine, West Virginia University, WV, USA; ^5^Division of Cardiology, Westchester Medical Center, Valhalla, NY, USA; ^6^Division of Cardiology, Rush University Medical Center, Chicago, IL, USA; ^7^Division of Cardiology, West Virginia University, Morgantown, WV, USA

## Abstract

**Objectives:**

To compare percutaneous mitral valve repair (PMVR) with optimal medical therapy (OMT) in patients with heart failure (HF) and severe functional mitral regurgitation (FMR).

**Background:**

Many patients with HF and FMR are not suitable for surgical valve replacement and remain symptomatic despite maximal OMT. PMVR has recently emerged as an alternative solution.

**Methods:**

We performed a systematic review and a meta-analysis to address this question. Cochrane CENTRAL, MEDLINE, and Scopus were searched for randomized (RCT) and nonrandomized studies comparing PMVR with OMT in patients with HF and FMR. Primary endpoint was all-cause midterm mortality (at 1 and 2 years). Secondary endpoints were 30-day mortality and cardiovascular mortality and HF hospitalizations, at maximum follow-up. Studies including mixed cohort of degenerative and functional MR were allowed initially but were excluded in a secondary sensitivity analysis for each of the study's end points. This meta-analysis was performed following the publication of two RCTs (MITRA-FR and COAPT).

**Results:**

Eight studies (six observational, two RCTs) comprising 3,009 patients were included in the meta-analysis. In comparison with OMT, PMVR significantly reduced 1-year mortality (RR: 0.70 [0.56, 0.87]; p=0.002; I^2^=47.6%), 2-year mortality (RR: 0.63 [0.55, 0.73]; p<0.001; I^2^=0%), and cardiovascular mortality (RR: 0.32 [0.23, 0.44]; p<0.001; I^2^=0%). No significant difference between PMVR+OMT and OMT was noted in HF hospitalization (HR: 0.69 [0.40, 1.20]; p=0.19; I^2^=85%) and 30-day mortality (RR: 1.13 [0.68, 1.87]; p=0.16; I^2^=0%).

**Conclusions:**

In comparison with OMT, PMVR significantly reduces 1-year mortality, 2-year mortality, and cardiovascular mortality in patients with HF and severe MR.

## 1. Introduction

Functional mitral regurgitation (FMR) is seen in most patients with heart failure (HF) and is classified as moderate to severe in 30% of them [1, 2]. The presence of significant FMR in patients with left ventricular dysfunction is associated with adverse outcomes, including death and frequent hospitalization for HF [3–5]. Optimal medical therapy (OMT) may provide symptomatic relief in some patients but many remain symptomatic despite maximal OMT [6]. Contemporary surgical mitral repair and replacement operations are performed with excellent short-term outcomes. However, only a minority of patients with FMR are referred for isolated mitral valve repair or replacement due to the lack of compelling data proving the long-term efficacy of surgical interventions for FMR [7, 8]. The emergence of percutaneous mitral valve repair (PMVR) was accompanied with a wealth of clinical investigations aiming to assess its value in addressing the unmet need of treating severe symptomatic FMR in HF patients [9, 10]. Several studies have demonstrated the safety and efficacy of the MitraClip (Abbott Vascular, Lake Bluff, Illinois) in patients with FMR [11–13]. However, only a few studies compared the outcomes of PMVR with MitraClip to OMT. We hence performed a systematic review and a meta-analysis to address these questions.

## 2. Methods

This meta-analysis was performed in accordance with the Preferred Reporting Items for Systematic review and Meta-Analyses (PRISMA) guidelines and the American Heart Association guidelines [14]. We utilized the relevant keywords “MitraClip”, “percutaneous mitral valve repair”, and “transcatheter mitral valve repair” in conjunction with MeSH terms to search MEDLINE, Cochrane, CENTRAL, and Scopus databases. The search was conducted from inception of the databases to September 25, 2018 (Supplemental Table 1). A supplementary search was done using citation chasing from relevant articles and hand searching of journals. Sources for the supplementary search included bibliographies of relevant reviews, editorials from major medical journals, websites of major journals, and conference proceedings for indexed abstracts. No language restrictions were placed.

All retrieved articles were transferred to EndNote X7 (Clarivate Analytics, Pennsylvania, United States) and duplicates were identified and removed. The remaining articles were screened by 2 reviewers (MUL and MSU) based on title and abstract. A third reviewer (MSK) was consulted to resolve discrepancies. Articles were selected based on the following eligibility criteria: (I) PMVR was compared with OMT in adult population (age ≥18 years) and at least 70% of the patients had heart failure complicated by functional MR. Study data were sought from the full texts of the included articles. Data were abstracted on study characteristics, baseline variables of patients, and outcomes of interest. In case cohorts of patients overlapped between studies, we included the study with the larger sample size in the analysis. When available, data from propensity-matched cohorts was preferred over unmatched data. The primary outcome was midterm all-cause mortality measured at 1- and 2-year intervals. The secondary outcomes were 30-day mortality, HF related hospitalizations, and cardiovascular death.

Risk of bias was assessed by two independent reviewers (MSU and MAAK), and a third reviewer was consulted to solve disagreements. Cochrane Collaboration's risk of bias 2.0 (ROB 2.0) tool was used to ascertain the risk of bias of the RCTs while the “Risk of Bias In Nonrandomized Studies-of Interventions” (ROBINS-I) tool was used to assess the risk of bias of observational studies.

Review manager (v.5.3) and Open MetaAnalyst were used to perform the analysis. For the mortality outcomes, odd ratio and 95% confidence intervals (CIs) were calculated using raw, unadjusted data from each included study. For HF hospitalization, the hazard ratios (HRs) provided by the studies were converted to generic inverse variances and standard errors and used as the effect size. The ORs/HRs were pooled using a random-effects model because of anticipated heterogeneity. Subgroup analysis according to type of study (observational versus RCTs) was conducted, and the chi-squared test was used to evaluate subgroup differences. Leave-one-out sensitivity analysis was conducted for all outcomes to assess if any single study disproportionately influenced the results. In order to study a cohort exclusively composed of patients with functional MR, we conducted a sensitivity analysis by removing studies with both functional MR and degenerative MR patients. Furthermore, we carried out a cumulative meta-analysis on primary outcome to study temporal trends. This chronological meta-analysis reveals if there is a consistency in the results of consecutive studies and indicates the point at which no further studies are necessary because the results continually favor 1 intervention. A secondary analysis was conducted to estimate the pooled risk difference between the PMVR and OMT groups per 1000-patient years and subsequently calculate the Number Needed to Treat (NNT) to prevent mortality. Heterogeneity across studies was evaluated using the I^2^ index, and a value of I^2^=25%-50% was considered mild, 50%-75% moderate, and >75% severe. Visual inspection of the funnel plot and Egger's regression test were used to assess publication bias. A p value of <0.05 was considered significant in all cases.

## 3. Results

**Figure 1:**
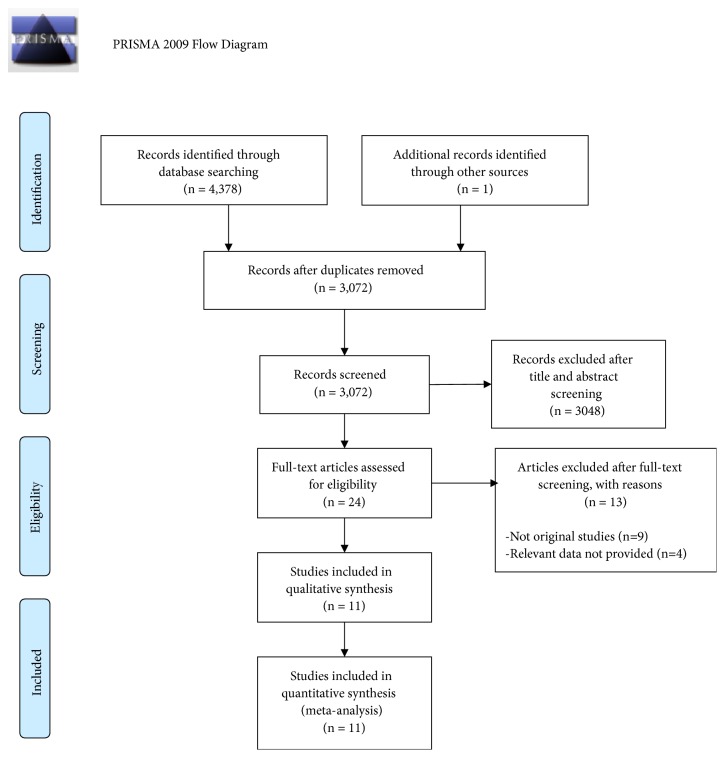
Study flow chart.

The initial search revealed 4,379 potentially relevant articles. After excluding duplicates and nonrelevant or incomplete publications (abstracts), 8 primary studies including 3,009 patients (1,689 in the PMVR arm, and 1320 in the OMT arm) were used in the synthetic analysis (Figure 1) [15–22].

### 3.1. Quality Assessment

All included observational studies were of moderately good methodological quality (Supplementary Tables 2 and 3). Five of the observational studies used propensity-matched analysis. Although both included RCTs had a robust methodology, there was a risk of bias due to lack of allocation concealment and lack of blinding in these studies.

### 3.2. Patient and Study Characteristics

The average age of the included patients was 72 years, and 62% of them were male. The average LVEF of the population was 33%, and 69% of them were classified as New-York-Heart-Association class III or IV. More than half (53%) of the study population had been diagnosed with coronary artery disease, and 48% had a history of atrial fibrillation. Baseline characteristics are outlined in Table 1. A summary of the inclusion criteria and study characteristics are given in Table 2.

### 3.3. Meta-Analysis of 1-Year Mortality

**Figure 2:**
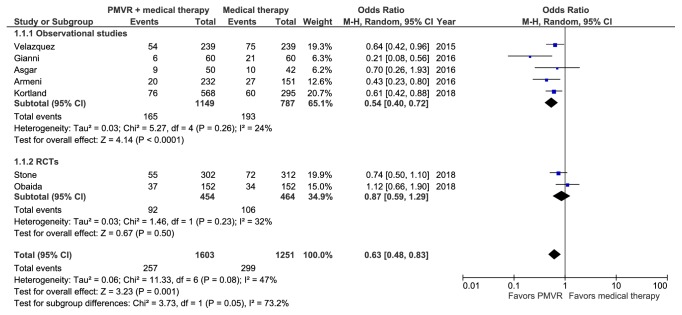
Forest plot displaying the risk of 1-year mortality in the PMVR group compared to the OMT group.

Seven studies representing 2,854 patients reported all-cause mortality at 1 year [15, 16, 18–21, 23]. A meta-analysis of these studies showed that PMVR significantly reduced 1-year all-cause mortality in comparison with OMT (RR: 0.70 [0.56, 0.87]; p=0.002; I^2^=47.6%) (Figure 2). Observational studies corroborated the overall finding, showing significant change in relative risk of 0.61 ([0.48, 0.78]; p<0.001; I^2^=26.3%). In contrast the results from RCTs were nonsignificant (RR: 0.90 [0.66, 1.23]; p=0.51; I^2^=33.3%). However, the difference between the two subgroups was nonsignificant (p interaction >0.05). Sensitivity analysis by removing studies including degenerative MR patients did not significantly change the results (RR: 0.76 [0.59, 0.99]; p=0.043; I^2^=48.8%).

### 3.4. Meta-Analysis of 2-Year Mortality

**Figure 3:**
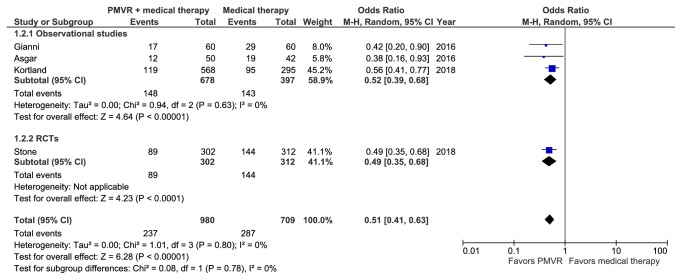
Forest plot displaying the risk of 2-year mortality in the PMVR group compared to the OMT group.

Four studies including 1,689 patients reported all-cause mortality rates at 2 years [16, 19, 21, 23]. A meta-analysis of these studies showed that PMVR was superior to OMT alone in reducing 2-year mortality (RR: 0.63 [0.55, 0.73]; p<0.001; I^2^=0%) (Figure 3). Both observational studies (RR: 0.63 [0.51, 0.76]; p<0.001; I^2^=0%) and the single RCT (RR: 0.64 [0.52, 0.79]; p<0.001) corroborated with the overall result (p interaction > 0.05). Sensitivity analysis removing studies that included patients with degenerative MR did not significantly change the results (RR: 0.47 [0.35, 0.62]; p<0.001; I^2^=0%).

### 3.5. Meta-Analysis of 30-Day Mortality

**Figure 4:**
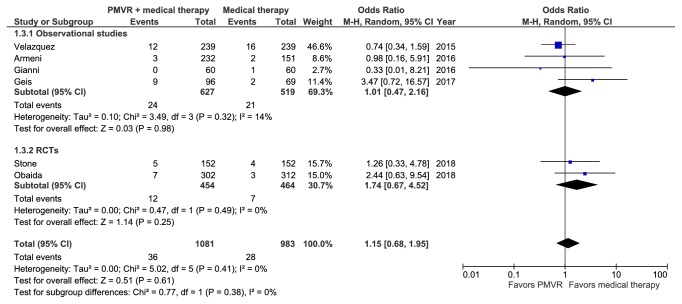
Meta-analysis displaying the risk of 30-day mortality in the PMVR group compared to the OMT group.

Six studies comprising 2,064 patients reported all-cause mortality at 30 days [15, 17, 18, 20–22]. A meta-analysis of these studies showed no significant difference between the PMVR and OMT groups (RR: 1.13 [0.68, 1.87]; p=0.16; I^2^=0%) (Figure 4). Results from both observational studies (RR: 1.0 [0.49, 2.02]; p=0.42; I^2^=11.4%) and RCTs (RR: 1.72 [0.66, 4.36]; p=0.26; I^2^=0%) were nonsignificant (p interaction >0.05). Sensitivity analysis by removing studies comprising patients with degenerative MR did not significantly change the results (RR: 1.38 [0.62, 3.07]; p=0.43; I^2^=0%).

### 3.6. Meta-Analysis of Cardiovascular Mortality

**Figure 5:**
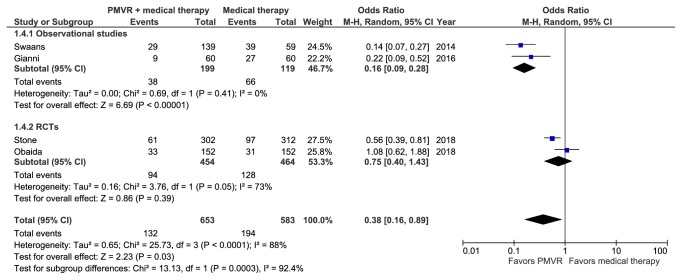
Forest plot displaying the risk of cardiovascular mortality in the PMVR group compared to the OMT group.

Four studies (representing 1,236 patients) reported cardiovascular mortality [15, 17, 20, 21]. During a mean follow-up of 1.54 years, PMVR significantly reduced cardiovascular mortality in comparison to OMT (RR: 0.53 [0.31, 0.91]; p=0.021; I^2^=85.6%) (Figure 5). Pooled observational studies also showed significant reduction in cardiovascular mortality (RR: 0.32 [0.23, 0.44]; p<0.001; I^2^=0%). Pooled RCTs, on the other hand, did not show significant reduction (RR: 0.81 [0.50, 1.31]; p=0.38; I^2^=71.5%) (p interaction < 0.05). The results became nonsignificant (RR: 0.65 [0.38, 1.09]; p=0.10; I^2^=76.7%) upon removing studies, which included patients with degenerative FMR.

### 3.7. Meta-Analysis of HF Hospitalizations

**Figure 6:**
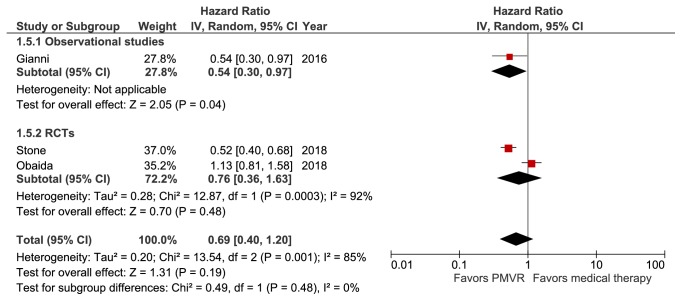
Forest plot displaying the incidence of heart failure hospitalization in the PMVR group compared to the OMT group.

Three studies (representing 1,038 patients) reported HF hospitalization at a mean follow-up of 1.64 years [20, 21, 23]. None of the studies included patients with degenerative MR. There was no significant difference in the incidence of HF hospitalization between patients treated with PMVR + OMT versus those who were treated with OMT alone (HR: 0.69 [0.40, 1.20]; p=0.19; I^2^=85%) (Figure 6). The difference between results from the observational study (HR: 0.54 [0.30, 0.97]; p=0.04) and RCTs (HR: 0.76 [0.36, 1.63]; p=0.48; I^2^=92%) was nonsignificant (p>0.05).

### 3.8. Pooled Risk Difference and Number Needed to Treat

The risk difference for all-cause mortality was -61.3 events per 1000-patient years and a Number Needed to Treat of nine to prevent one death per year. The risk difference for cardiovascular mortality was -53.7 events per 1000-patient years, with a Number Needed to Treat of five to prevent one cardiovascular death per year (Supplementary Table 4).

### 3.9. Leave-One-Out Meta-Analysis

The results for 30-day, 1-year, and 2-year mortality were robust, with no single study having a disproportionate effect on the results (Supplementary Figures 1-3). The cardiovascular mortality outcome became nonsignificant upon removal of all studies except one (Obadia, 2018) (Supplementary Figure 4).

### 3.10. Cumulative Meta-Analysis

A temporal trend towards higher mortality with PMVR (higher RRs) was seen for the 30-day mortality outcome. Lack of consistency for this outcome highlights the uncertainty of current evidence. For 1-year, 2-year, and cardiovascular mortality, no clear temporal shift in the results was seen (Supplementary Figures 5-8).

### 3.11. Publication Bias

The funnel plot (Supplementary Figure 9) suggested presence of publication bias. The vacant right, lower quadrant suggested that missing studies would have been of small size and could have possibly shown increased mortality with PMVR. Presence of publication bias was confirmed by Eggers regression test (p=0.009).

## 4. Discussion

This meta-analysis of more than 3000 patients with HF and severe MR shows that PMVR significantly reduces midterm all-cause and cardiovascular mortality compared with OMT alone. However, HF hospitalizations and 30-day mortality were not significantly different between the two groups. A previous meta-analysis has shown a similar reduction in all-cause mortality but also showed a reduction in HF hospitalizations with PMVR [23]. Nonetheless, this study was conducted prior to the publication of two recent RCTs (MITRA-FR and COAPT) [20, 21]. Our updated meta-analysis significantly enhances the evidence from the previous one, with an additional 888 patients included from one observational and two RCTs.

Functional mitral regurgitation results from left ventricular dilation and/or regional wall dyskinesis leading to dislocation of the papillary muscles and tethering of the leaflets. The differential negative impact of FMR on patient's symptomatology, progressive remodeling, and long-term outcomes of patients with HF has been long established [24, 25]. However, the ideal treatment for patients with FMR who remain symptomatic despite OMT has been an area of intense debate. Surgical mitral valve repair or replacement can successfully eliminate FMR, but neither intervention has been shown to reduce the morbidity and mortality associated with FMR [26, 27]. In addition, both approaches are associated with significant early risk of death and postoperative complications [27–29]. The emergence of transcatheter mitral valve repair and replacements systems led to a plethora of investigations assessing their utility specifically in patients with FMR.

The MitraClip system is the first PMVR system to become commercially available. Although the MitraClip was initially approved to treat degenerative (primary) mitral regurgitation, many FMR patients were treated with the MitraClip on an off-label basis [12, 13]. This has led to a substantial body of evidence suggesting its safety and efficacy in this challenging group of patients. Nonetheless, data comparing PMVR to OMT remained limited. A previous meta-analysis addressing the same question found beneficial effects for PMVR in FMR patients but it only included observational data [23]. In light of the recent publication of the first landmark RCTs comparing OMT to PMVR, we sought to perform an updated systematic review and a meta-analysis to elucidate the best available evidence on the key question of whether PMVR carries an incremental benefit over OMT alone in patients with FMR. Our analysis revealed that PMVR with the MitraClip decreased all-cause mortality by 30% at 1 year and by 37% at 2 years. While the 1-year mortality benefit was mostly driven by observational data, the 2-year benefit was corroborated in both observational studies and the single RCT reporting 2-year mortality data. In addition, PMVR was associated with lower cardiovascular death and no excess short-term mortality (at 30 days).

The findings of this meta-analysis raise several important issues: (1) FMR is not a one-size-fits-all entity. This is best illustrated by the striking differences between the COAPT and MITRA-FR RCTs. Both of these trials were set to address the same questions (utility of PMVR in FMR) and reached strikingly different conclusions. This is likely because each study enrolled a different subset of FMR patients. The MITRA-FR trial enrolled patients with severely dilated ventricles (no limit of left ventricular dimensions) and less degrees of FMR (effective regurgitant orifice area>20 mm^2^, regurgitant volume >30 ml/beat), while COAPT only allowed patients with less dilated ventricles (left ventricular diastolic dimension <7 cm) and higher degrees of FMR (effective regurgitant orifice area>30 mm^2^, regurgitant volume >45 ml/beat). In other words, COAPT likely selected patients in whom the valvular disease was a large component of their pathology while the valve disease was likely a pure bystander in MITRA-FR. Hence, the substantial benefits of PMVR observed in COAPT versus MITRA-FR are not surprising. (2) The underlying mechanism by which PMVR substantially improved the outcomes of FMR patients remains a subject of study. However, this meta-analysis might confirm the earlier observations suggesting a major role of PMVR-induced reverse remodeling in improving long-term outcomes of patients with FMR [12, 30]. (3) The mortality benefit that was observed in our meta-analysis was robust but was more consistent at 2 years (I^2^=0), suggesting that longer-term follow-up might be needed in future PMVR investigations to elucidate the potential benefit of the therapy in FMR patients. (4) The lack of reduction in HF hospitalization should be interpreted with caution, due to the limited number of studies reporting this endpoint and the potential subjectivity of this endpoint itself.

Limitations: certain limitations must be taken into consideration when interpreting the results of this study. Firstly, the results of this analysis were partially based on observational studies, which are relatively more susceptible to bias due to confounding. It must be noted, however, that we found all observational studies to be of robust methodological quality, with most employing propensity-matched analysis. Second, the results for the cardiovascular mortality and HF hospitalization outcomes had significant heterogeneity, which could not be explained by subgroups according to study design. Third, using RRs stratified according to time (1 and 2 years) for the mortality outcome could potentially lead to an overestimation of the effect size when compared to time-to-event effect sizes. Fourth, variation in follow-up time was not accounted for in the cardiovascular mortality outcome, which could have led to some bias.

## 5. Conclusions

This meta-analysis suggests that, in comparison with OMT, PMVR is not associated with excess 30-day mortality and significantly reduce all-cause mortality at 1 and 2 years. Given the heterogeneity in the included FMR populations, further studies are needed to confirm the results of this meta-analysis and to identify to ideal candidate for PMVR.

## Figures and Tables

**Table 1:** Baseline characteristics of included patients.

Author (year)	Average Age	Average MR Grade	Male Sex %	Previous Afib %	NYHA III-IV (%)	Average LVEF %
	Overall (Treatment/Control)		Overall (Treatment/Control)	Overall (Treatment/Control)	Overall (Treatment/Control)	Overall (Treatment/Control)
Velazquez, 2015	73.7 (73.7)	3-4+	57.1 (59.8/54.4)	33 (33/33)		33.21 (34/32)
Asgar, 2016	71.8 (75/68)	3-4+	75.37 (74/77)	39 (35/43)	74.5 (73/76)	36 (37/35)
Armeni, 2016	71 (71/71)		73.39 (73/74)	49.48 (53/40.7)	87.87 (88.5/86.4)	36.11 (36.8/34.5)
Giannini, 2016	75.5 (75/76)	3-4+	66.5 (70/63)	61.55 (64.9/58.2)	79 (78.2/79.8)	41.75 (41.5/42)
Geis, 2017	62.01 (68.2/54.3)		41.57 (74.4/0.65)	58.82 (61.6/52.8)		54.65 (54.4/55.2)
Obadia, 2018	70.35 (70.1/70.6)	3-4+	74.65 (78.9/70.4)	51.29 (55.6/43)	56.8 (86.3/61.9)	36.07 (37.22/33.85)
Kortlandt, 2018	74.02 (73.96/74.15)	3-4+	54.93 (56.5/51.9)	46.3 (65.0/23)		25.45 (25/26)
Stone, 2018	72.26 (71.7/72.8)	3-4+	64.01 (66.6/61.5)	55.22 (57.3/53.2)	60.86 (57/64.6)	31.3 (31.3/31/3)

MR: mitral regurgitation; Afib: atrial fibrillation; NYHA: New York Heart Association; LVEF: left ventricular ejection fraction.

**Table 2:** Characteristics of the included studies.

Author (year)	Total Participants	Participants	Participants	Follow-up, weeks	Primary endpoints
	(# of FMR)	Treatment Group	Control Group		
Velazquez, 2015	478 (415 FMR)	239	239	48	All-cause mortality
Asgar, 2016	92 (45 FMR)	50	42	144	All-cause mortality; Cost-effectiveness metrics
Armeni, 2016	383 (383 FMR)	232	151	48	Cost-effectiveness metrics
Giannini, 2016	120 (NR)	60	60	48	All-cause mortality
Geis, 2017	155 (124 FMR)	86	69	48	Cardiac remodeling metrics (LVEF, LVESD)
Obaida, 2018	304 (304 FMR)	152	152	240	Composite: all-cause mortality and HF hospitalizations
Kortlandt, 2018	863 (593 FMR)	568	295	96	All cause mortality
Stone, 2018	614 (614 FMR)	302	312	96	HF hospitalizations at 24 months, device related complications at 12 months

FMR: functional mitral regurgitation; LVEF: left ventricular ejection fraction; LVESD: left ventricular end systolic diameter

## Data Availability

All the data used in the analysis is presented within the manuscript.

## Supplementary Materials

Supplementary Materials Supplementary Table S1: search strategy used in each database searched. Supplementary Table S2: Quality Assessment of the Observational Studies using the Robins-1 tool. Supplementary Table S3: Quality Assessment of the Randomized Studies using the ROB 2.0 scale. Supplementary Table S4: Pooled Analysis for the Number Needed to Treat. Supplementary Figure-1: leave-one-out meta-analysis for the 1-year mortality outcome. Supplementary Figure-2: leave-one-out meta-analysis for the 2-year mortality outcome. Supplementary Figure-3: leave-one-out meta-analysis for the 30-day mortality outcome. Supplementary Figure-4: leave-one-out meta-analysis for the cardiovascular mortality outcome. Supplementary Figure-5: cumulative meta-analysis for the 1-year mortality outcome. Supplementary Figure-6: cumulative meta-analysis for the 2-year mortality outcome. Supplementary Figure-7: Cumulative meta-analysis for the 30-day mortality outcome. Supplementary Figure-8: Cumulative meta-analysis for the cardiovascular mortality outcome. Supplementary Figure-9: funnel plot for the heterogeneity of the included studies.
