# Supplementary material for: Percutaneous Mitral Valve Repair versus Optimal Medical Therapy in Patients with Functional Mitral Regurgitation: A Systematic Review and Meta-Analysis
Source: J Interv Cardiol. 2019 Apr 21;2019:2753146. doi: 10.1155/2019/2753146 (PMC6739764; doi:10.1155/2019/2753146)

**Online Supplementary Material**

| Database | Search Strategy | Articles retrieved |
| --- | --- | --- |
| MEDLINE | (Percutaneous[All Fields] AND ("mitral valve"[MeSH Terms] OR ("mitral"[All Fields] AND "valve"[All Fields]) OR "mitral valve"[All Fields]) AND ("wound healing"[MeSH Terms] OR ("wound"[All Fields] AND "healing"[All Fields]) OR "wound healing"[All Fields] OR "repair"[All Fields])) OR mitraclip[All Fields] OR (transcatheter[All Fields] AND ("mitral valve"[MeSH Terms] OR ("mitral"[All Fields] AND "valve"[All Fields]) OR "mitral valve"[All Fields]) AND ("wound healing"[MeSH Terms] OR ("wound"[All Fields] AND "healing"[All Fields]) OR "wound healing"[All Fields] OR "repair"[All Fields])) | 1,586 |
| Cochrane CENTRAL | (Percutaneous mitral valve repair OR mitraclip OR transcatheter mitral valve repair)ti:ab:kw | 120 |
| Scopus | (TITLE-ABS-KEY ( percutaneous AND mitral AND valve AND repair ) OR TITLE-ABS-KEY ( mitraclip ) OR TITLE-ABS-KEY ( transcatheter AND mitral AND valve AND repair ) ) | 2,672 |

**Supplementary Table S1: Search strategy used in each database searched**

| **Study** | | **Pre-intervention** | | **At intervention** | **Post-intervention** | | | | **Overall risk of bias** |
| --- | --- | --- | --- | --- | --- | --- | --- | --- | --- |
| **Author** | **Year** | **Bias due to confounding** | **Bias in selection of participants into the study** | **Bias in classification of interventions** | **Bias due to deviations from intended interventions** | **Bias due to missing data** | **Bias in measurement of outcomes** | **Bias in selection of the reported result** | **low/moderate/serious / critical** |
| Armeni et al | 2016 | low | low | low | low | low | moderate | low | moderate |
| Asgar et al | 2017 | low | low | low | low | low | moderate | low | moderate |
| Giannini et al | 2016 | low | low | low | low | moderate | low | moderate | moderate |
| Swaans et al | 2014 | moderate | moderate | low | low | moderate | low | low | moderate |
| Valazquez et al | 2015 | low | low | low | low | moderate | low | low | moderate |
| Kortlandt et al | 2018 | low | low | low | low | low | low | low | low |
| Whitlow et al | 2012 | moderate | moderate | low | low | moderate | low | low | moderate |
| Geiss et al | 2017 | moderate | moderate | low | low | low | low | low | moderate |

**Supplementary Table S2:**

**Quality Assessment of the Observational Studies using the Robins-1 tool**

| **Author** | **Year** | **Bias arising from the randomization process** | **Bias due to deviations from intended interventions** | **Bias due to missing data** | **Bias in measurement of outcomes** | **Bias in selection of the reported result** | **low/moderate/serious / critical** |
| --- | --- | --- | --- | --- | --- | --- | --- |
| Obadia | 2018 | low | moderate | moderate | low | low | low |
| Stone | 2018 | moderate | moderate | moderate | low | low | moderate |

**Supplementary Table S3:**

**Quality Assessment of the Randomized Studies using the ROB 2.0 scale**

| **Outcome** | **Studies** | **RD [95% CI] per 1000 patient-years** | **NNT** |
| --- | --- | --- | --- |
| **All-cause mortality** | OSs | -67.0 [-119.9, -14.1] | 9 |
|  | RCTs | -40.9 [-143.9, 62.2] | 13 |
|  | **Total** | **-61.6 [-108.6, 14.5]** | **9** |
| **CV mortality** | OSs | -316.6 [-549.3, -83.8] | 2 |
|  | RCTs | -38.1 [-94.9, 18.7] | 19 |
|  | **Total** | **-53.7 [-108.9, 1.5]** | **5** |

**Supplementary Table S4: Pooled Analysis for the Number Needed to Treat.**

CV (Cardiovascular); CI (Confidence Interval); NNT (Number Needed to Treat); OS (Observational Studies); RCTs (Randomized Controlled Trials); RD (Risk Difference)

**Supplementary Figures**

**Supplementary Figure-1: Leave-one-out meta-analysis for the 1-year mortality outcome.**

No single study had a disproportionate effect on the results.


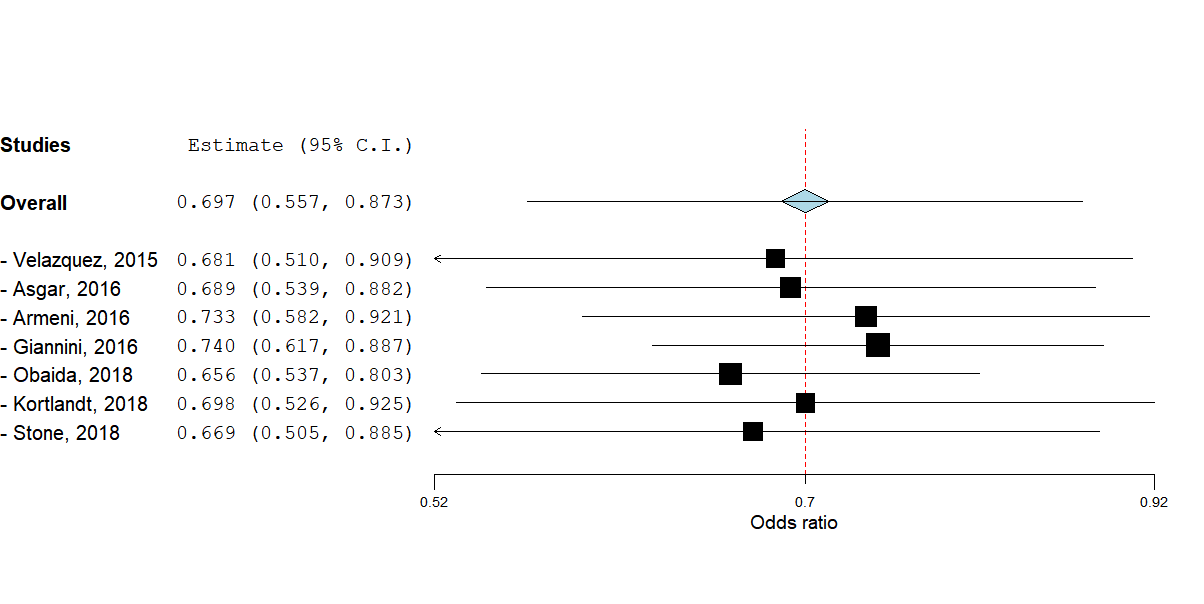


**Supplementary Figure-2: Leave-one-out meta-analysis for the 2-year mortality outcome.**

No single study had a disproportionate effect on the results.


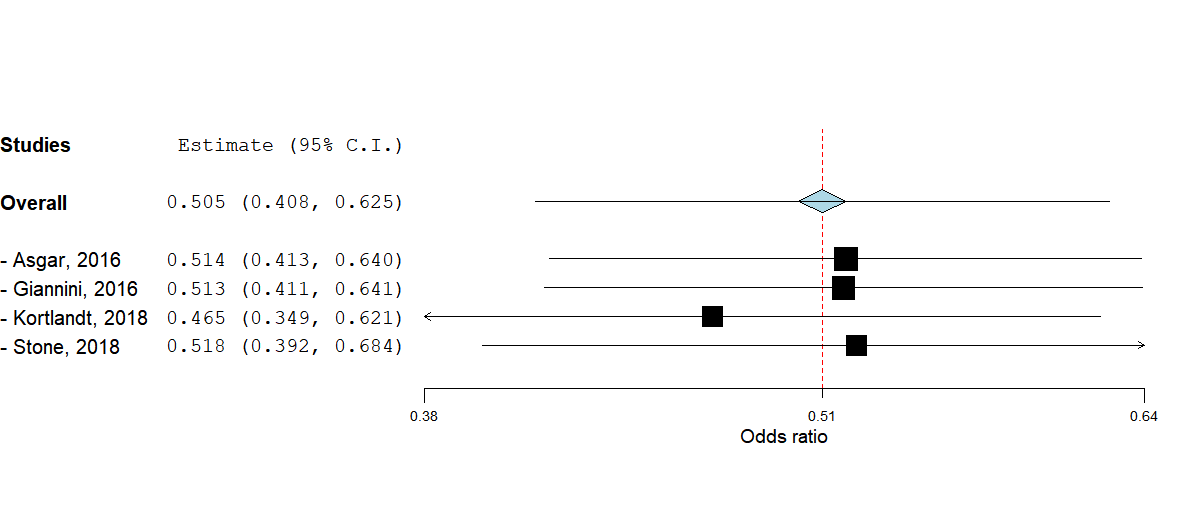


**Supplementary Figure-3: Leave-one-out meta-analysis for the 30-day mortality outcome.**

No single study had a disproportionate effect on the results.


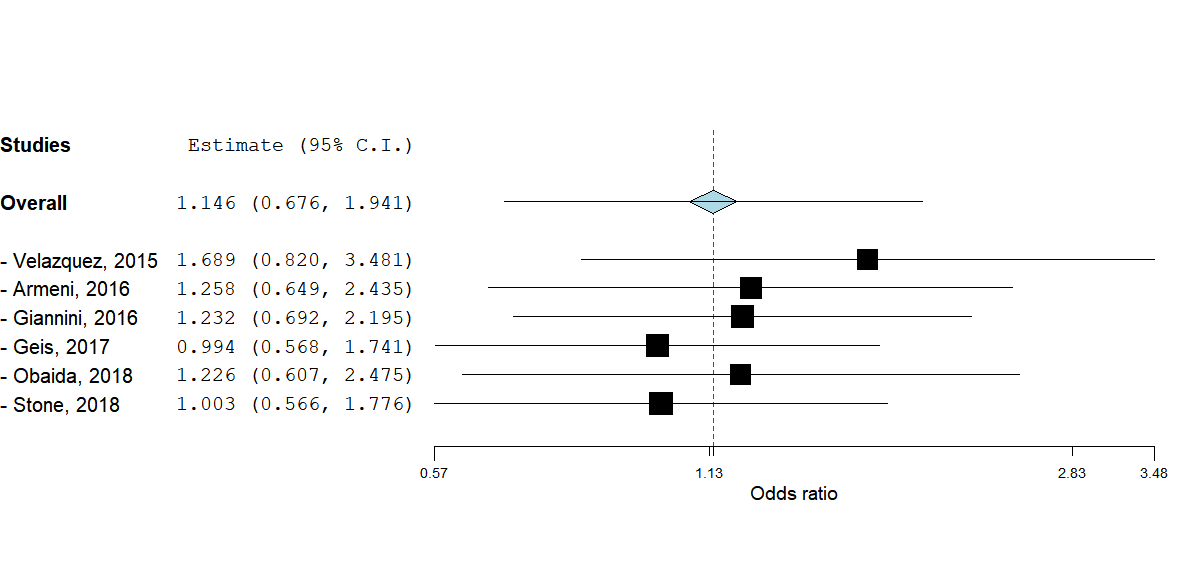


**Supplementary Figure-4: Leave-one-out meta-analysis for the cardiovascular mortality outcome.**

Removal of the study by Obadiah et al changes the outcome to non-significant

**
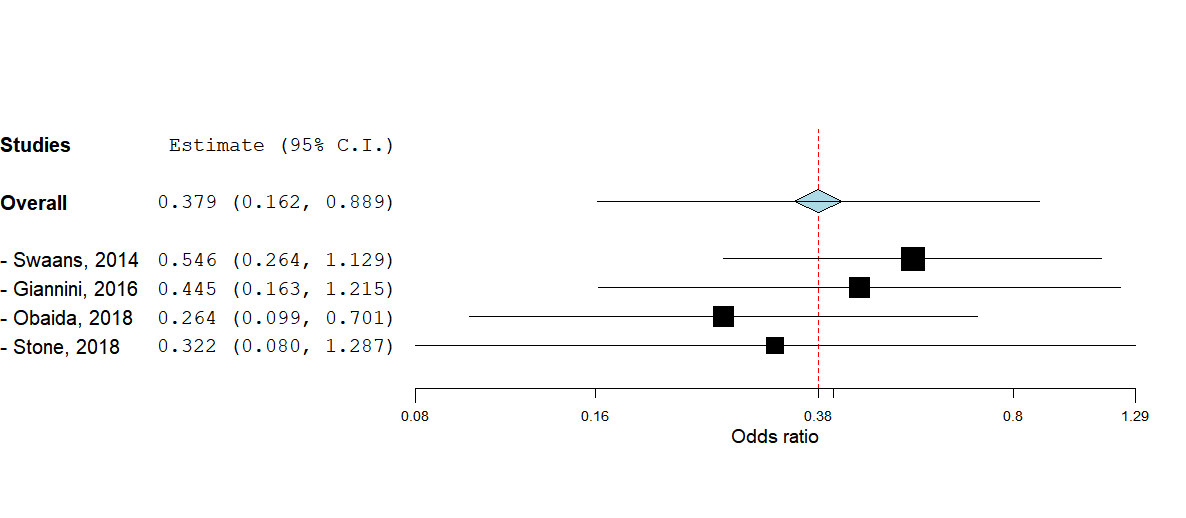
**

**Supplementary Figure-5: Cumulative meta-analysis for the 1-year mortality outcome.**

No temporal shift in results is noted


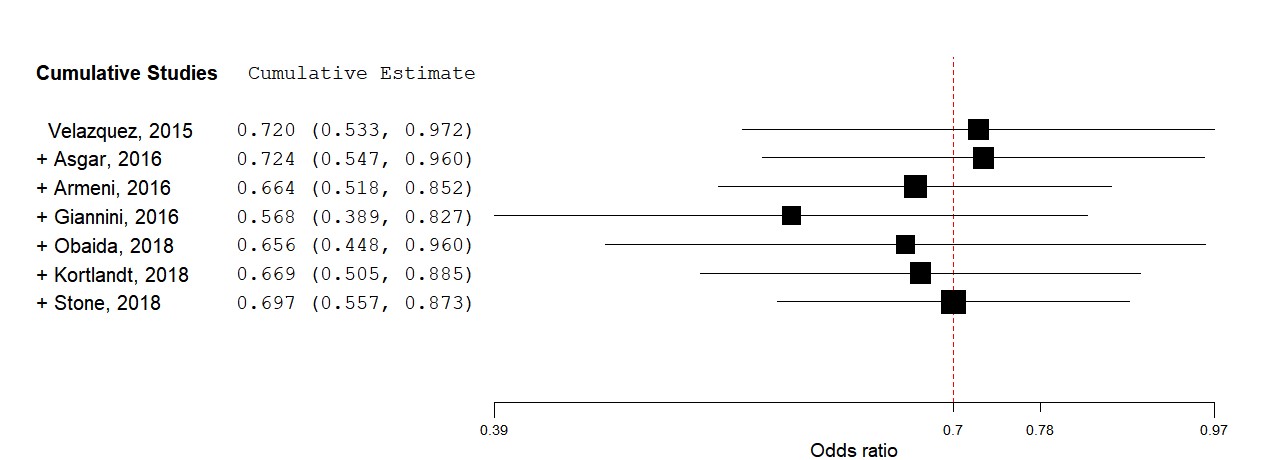


**Supplementary Figure-6: Cumulative meta-analysis for the 2-year mortality outcome.**

No temporal shift in results is noted


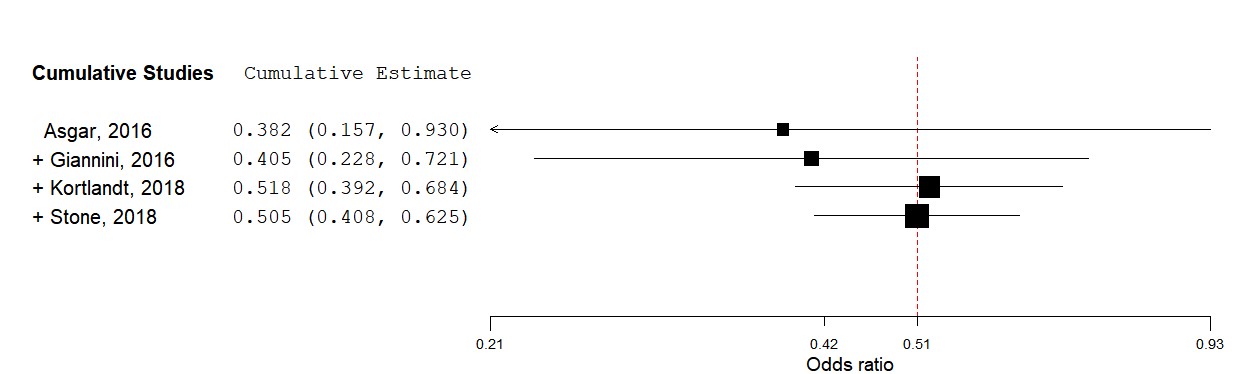


**Supplementary Figure-7: Cumulative meta-analysis for the 30-day mortality outcome.**A temporal trend towards higher mortality with PMVR (higher RRs) is seen


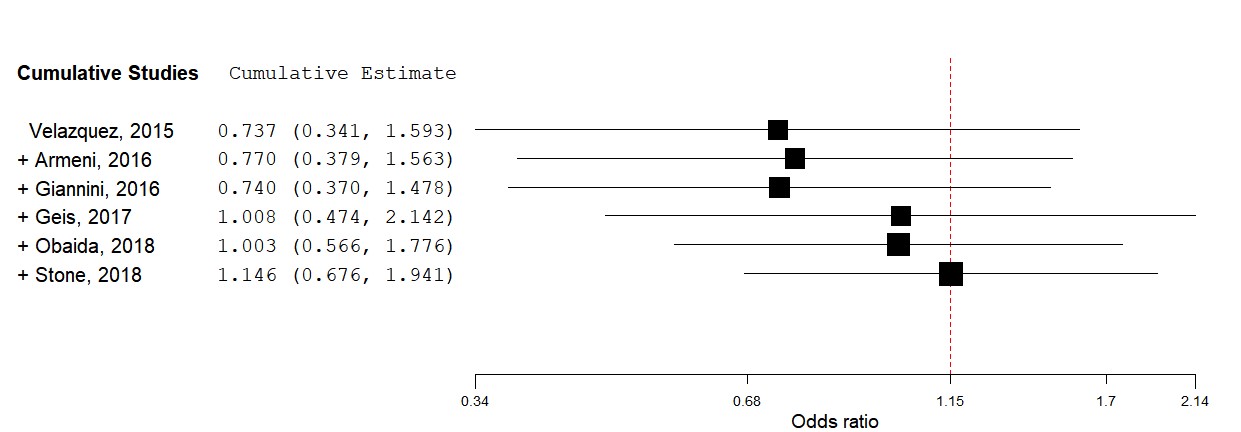


**Supplementary Figure-8: Cumulative meta-analysis for the cardiovascular mortality outcome.**

No temporal shift in results is noted


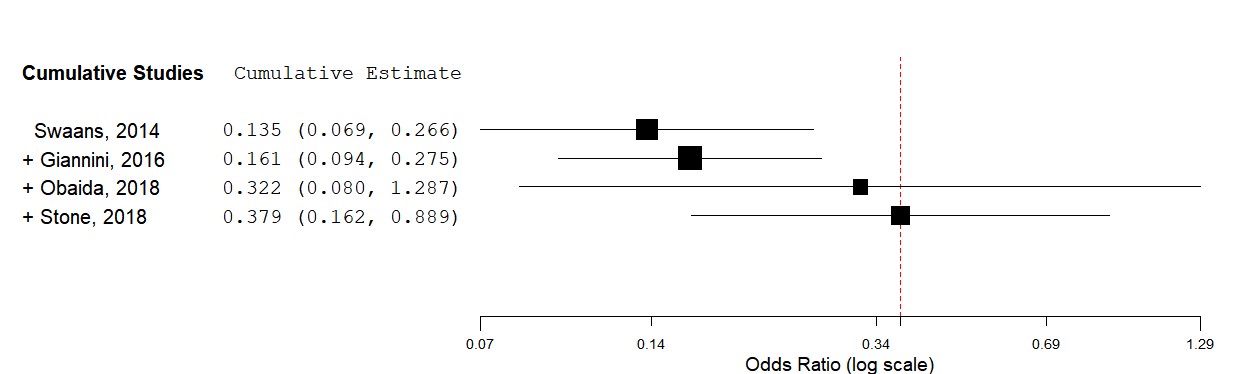


**Supplementary Figure-9: Funnel plot for the heterogeneity of the included studies**The funnel plot demonstrates likely presence of publication bias


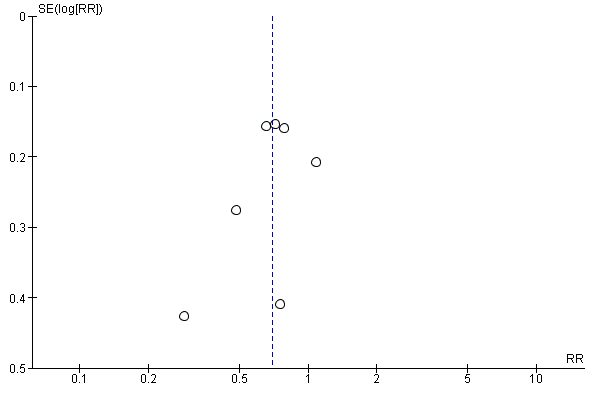

Supplement: Supplementary Materials — Supplementary Table S1: search strategy used in each database searched. Supplementary Table S2: Quality Assessment of the Observational Studies using the Robins-1 tool. Supplementary Table S3: Quality Assessment of the Randomized Studies using the ROB 2.0 scale. Supplementary Table S4: Pooled Analysis for the Number Needed to Treat. Supplementary Figure-1: leave-one-out meta-analysis for the 1-year mortality outcome. Supplementary Figure-2: leave-one-out meta-analysis for the 2-year mortality outcome. Supplementary Figure-3: leave-one-out meta-analysis for the 30-day mortality outcome. Supplementary Figure-4: leave-one-out meta-analysis for the cardiovascular mortality outcome. Supplementary Figure-5: cumulative meta-analysis for the 1-year mortality outcome. Supplementary Figure-6: cumulative meta-analysis for the 2-year mortality outcome. Supplementary Figure-7: Cumulative meta-analysis for the 30-day mortality outcome. Supplementary Figure-8: Cumulative meta-analysis for the cardiovascular mortality outcome. Supplementary Figure-9: funnel plot for the heterogeneity of the included studies. [file 2753146.f1.zip › 2753146 .f1/mat.2753146.v2.docx]
